# Cumulative Adverse Childhood Experiences and Adult Social Isolation: The Roles of Family Support and Strain

**DOI:** 10.1093/geroni/igaf122.2110

**Published:** 2025-12-31

**Authors:** Stephanie Robert, Jooyoung Kong, Weidi Qin

**Affiliations:** University of Wisconsin–Madison, Madison, Wisconsin, United States; University of Wisconsin–Madison, Madison, Wisconsin, United States; University of Wisconsin–Madison, Madison, Wisconsin, United States

## Abstract

**Background/objectives:**

Childhood trauma can have lasting effects on various aspects of adult life, one of which is the potential impairment in the ability to form healthy, stable relationships. Despite this, little research has explored the long-term effects of such trauma on social isolation in adulthood. This study investigates the association between adverse childhood experiences (ACEs) and adult social isolation, with a focus on how family support and strain may impact this relationship.

**Methods:**

Data were drawn from three-wave longitudinal surveys of the Midlife in the United States (MIDUS) study. Social isolation was measured using a composite scale that included partnership status, contact with family and friends, and participation in social activities. Growth curve modeling was used to assess the effects of ACEs, family support, and family strain on social isolation while controlling for covariates such as age, sex, race, education, and self-rated health.

**Results:**

ACEs were positively associated with social isolation, though the time effects of ACEs were not statistically significant. Both family support and strain were associated with lower levels of social isolation. For those with higher ACEs, the protective effect of family support was weakened. When excluding partnership status from the social isolation measure and including it as a covariate, most results remained consistent.

**Conclusions:**

ACEs contribute to increased social isolation in adulthood and may attenuate the protective role of family support in reducing social withdrawal. Further research is needed to identify underlying mechanisms, which could inform targeted interventions to promote social connectedness for adults with childhood trauma.

